# Lower serum levels of total cholesterol are associated with higher urinary levels of 8-hydroxydeoxyguanosine

**DOI:** 10.1186/1743-7075-10-59

**Published:** 2013-10-02

**Authors:** Hiroyuki Kikuchi, Akiko Nanri, Ai Hori, Masao Sato, Kazuaki Kawai, Hiroshi Kasai, Tetsuya Mizoue

**Affiliations:** 1 Department of Epidemiology and Prevention, Clinical Research Center, National Center for Global Health and Medicine, Shinjuku-ku, Tokyo 162-8655, Japan; 2 Department of Safety and Health, Tokyo Gas Co., Ltd., Minato-ku, Tokyo, Japan; 3 Laboratory of Nutrition Chemistry, Faculty of Agriculture, Kyushu University, Fukuoka City, Japan; 4 Institute of Industrial Ecological Sciences, Department of Environmental Oncology, University of Occupational and Environmental Health, Kitakyushu City, Fukuoka, Japan

**Keywords:** Oxidative stress, 8-hydroxydeoxyguanosine (8-OHdG), Epidemiologic studies, Cross-sectional studies, High-density lipoprotein (HDL-C), Low-density lipoprotein (LDL-C), Total cholesterol

## Abstract

**Background:**

Lower serum total (TC), high-density lipoprotein (HDL-C) and low-density lipoprotein cholesterols (LDL-C) have been linked to an increased risk of cancer in various sites, but its underlying mechanism remains unclear. In an attempt to clarify the association between cholesterol levels and oxidative DNA damage, we investigated the relationship between serum cholesterol and urinary 8-hydroxydeoxyguanosine levels in a Japanese working population.

**Methods:**

The study subjects were 294 men and 209 women aged 21-66 years in two Japanese municipal offices. Urinary 8-hydroxydeoxyguanosine (8-OHdG) was measured using an automated high-pressure liquid chromatography. Linear regression analysis was used to examine the associations of urinary 8-OHdG with TC, HDL-C and LDL-C levels with adjustment for sex, age, smoking and body mass index. Subgroup analyses were conducted by smoking status in men and age in women. Analysis of covariance was employed to estimate adjusted means of urinary 8-OHdG across TC category.

**Results:**

After multivariate adjustment, urinary 8-OHdG levels were inversely associated with serum TC levels (β = −0.0015, p < 0.05) and LDL-C levels (β = −0.0012, p = 0.07). The inverse association with TC was apparent among smoking men (β = −0.0017, p < 0.05) and among women aged less than 48 years (β = −0.0040, p < 0.01). 8-OHdG decreased as TC increased (up to 219 mg/dL); subjects with TC levels of <160 mg/dL had a 17.4% higher adjusted mean of 8-OHdG than did those with TC levels of 200–219 mg/dL.

**Conclusion:**

Results suggest that circulating low TC levels are associated with higher oxidative DNA damage.

## Introduction

Lower circulating total (TC) [1-3], high-density lipoprotein (HDL-C) [4] and low-density lipoprotein cholesterols (LDL-C) [5] have been shown to be associated with an increase risk of total cancer, as well as cancer of the lung, prostate, stomach or colon [6,7]. Although these observed associations have been ascribed to the presence of preclinical cancer that decreases cholesterol levels [8,9], the association remained significant in several studies even after excluding cancer incidence that occurred during early period of follow up [2,6]. Thus, controversy continues whether low cholesterol increases cancer risk.

A clue to this question may be obtained from biomarker study linking low cholesterol to carcinogenesis in a healthy population. Oxidative DNA stress is thought to play a major role in carcinogenesis [10]. 8-hydroxydeoxyguanosine (8-OHdG) is a reliable marker of oxidative DNA damage and its concentrations in urine have been shown to predict cancer risk [11]. To our knowledge, only two studies examined the association between urinary 8-OHdG and serum cholesterol and their results are conflicting. Sakano et.al. reported a positive association between LDL-C and urinary 8-OHdG and no association between TC and 8-OHdG [12]. In contrast, Miyamaoto et al. found no apparent association between TC/HDL-C and urinary 8-OHdG [13]. These results, however, may be limited due to the use of ELISA method in measuring 8-OHdG, which is less accurate than the high performance liquid chromatography (HPLC) method [14].

We hypothesized that oxidative DNA damage would be increased in persons with low TC/HDL-C/LDL-C levels. Here, we examined the association between these lipids and urinary 8-OHdG levels measured by using HPLC in an apparently healthy working population.

## Methods

### Study subjects

**Figure 1:**
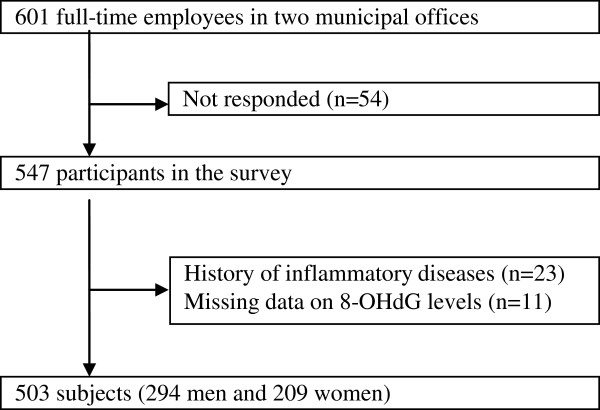
Flowchart of study protocol.

Figure 1 shows flowchart of study protocol. In July and November, 2006, a health survey was conducted among municipal employees of two offices in northeastern Kyushu, Japan, as described elsewhere [15]. Full-time employees were invited to participate in the survey (n = 601) and 547 responded (age range, 21-67 years; response rate, 91.0%). Study participants were asked to fill out the survey questionnaire beforehand, which were checked by research staff for completeness. Also obtained were data that were routinely collected in the health examination, including job title, anthropometric measurements, biochemical data, and information about medical history, smoking, physical activity and alcohol drinking.

We excluded subjects with a history of cancer (n = 8), cardiovascular disease (n = 4), cerebrovascular diseases (n = 6) or diabetes (n = 12), subjects who were currently under the care of physician due to chronic liver disease (n = 2) or nephritis (n = 1), subjects who were receiving medication for hyperlipidemia (n = 3) and pregnant women (n = 2). We further excluded subjects who had missing information on any of the variables used in the present analysis. Some participants had two or more conditions for exclusion. After exclusion, 503 (294 Japanese men and 209 women) were eligible for analysis. The protocol of the health study was approved by the ethics committee of the National Center for Global Health and Medicine, and the written informed consent was obtained from each subjects.

### Measurement of serum cholesterol levels and urinary 8-OHdG

During the health checkup, blood and urine samples were obtained. Serum TC, HDL-C and triglycerides were measured by using Quick auto neo T-Cho (Shino-Test Co., Tokyo), Cholestest N HDL (Daiichi Kagaku-Co., Tokyo), Wako L-Type (Wako Co. Tokyo), respectively. Among participants with fasting blood samples (n = 481), LDL-C was estimated according to the Friedewald formula: [LDL-C = TC-HDL-C-triglycerides/5]. Urinary 8-OHdG and creatinine were determined by HPLC method [14]. The accuracy of the measurement, estimated from the recovery of an added 8-OHdG standard, was 90-98%. When the same urine sample was analyzed three times, the variation of the data was within 7%.

### Data analysis

For continuous variables, normality was checked before statistical analysis. 8-OHdG levels were adjusted for urinary creatinine levels and log-transformed for association analyses. Multiple linear regression analyses were used to examine the associations of urinary 8-OHdG with TC, HDL-C and LDL-C levels while adjusting for sex, age (continuous), smoking (smoker or nonsmoker including past smoker) and body mass index (BMI) (kg/m^2^, continuous). To test the interaction, we added an interaction term of sex and each TC, HDL-C, LDL-C levels to the model. Subgroup analyses were conducted by smoking status in men (smoker and nonsmoker) and age group in women (<48 and ≥48 years, the median age of menopause in Japanese women [16]). Due to few female smokers (n = 4), subgroup analysis by smoking status was not performed in women.

After subjects were divided into seven groups according to TC concentrations (<160, 160-179, 180-199, 200-219, 220-239, 240-259 or ≥260 mg/dL), analysis of covariance was employed to estimate the means of log-transformed 8-OHdG and their standard errors. The means and their 95% confidence limits in log-scale were then back-transformed. A trend test was performed by treating TC as a continuous variable in a multivariable regression analysis. We repeated the above analyses by adding other covariates, including physical activity, alcohol consumption, hypertension status and job-title. All statistical tests were two-tailed and considered to be statistically significant at the 0.05 level. All analyses were done with Stata, version 9.1.

## Findings

The 294 subjects were men (58.4%), the mean age was 42.4 years old (range 21-66), and mean (±SD) BMI was 22.5 ± 3.5 kg/m^2^. As shown in Table 1, age and BMI were positively correlated with TC levels. Mean (SD) TC was 206 ± 33 mg/dL (men: 209 ± 33 mg/dL; women: 201 ± 34 mg/dL), HDL-C was 61.5 ± 16 mg/dL (men: 55.7 ± 14 mg/dL; women: 69.8 ± 15 mg/dL) and LDL-C was 122 ± 30 mg/dL (men: 125 ± 30 mg/dL; women: 117 ± 30 mg/dL). Median (interquartile range) 8-OHdG concentration was 3.01 (2.37, 4.03) μg/g creatinine (men: 3.09 [2.43, 3.97]; women; 2.99 [2.24, 4.21]), ranging from 0.8 to 10.0 μg/g creatinine.

**Table 1:** Characteristics of study subjects by serum total cholesterol concentrations

	**Serum total cholesterol concentrations (mg/dL)**							**Total**
	**<160**	**160-179**	**180-199**	**200-219**	**220-239**	**240-259**	**≥260**	
n	37	72	106	122	85	48	33	503
Men subjects,%	51	49	47	66	66	73	55	58
Age (S.D.), y	36 (10)	37 (9)	41 (12)	43 (9)	46 (10)	48 (9)	49 (9)	42 (11)
BMI (S.D.), kg/m^2^	21.5 (3.3)	21.1 (2.6)	21.8 (3.3)	22.6 (3.7)	23.4 (3.5)	24.1 (3.5)	24.1 (3.5)	22.5 (3.5)
Current Smoker,%	24	22	23	31	25	42	30	27

*BMI* Body mass index.

Table 2 shows the results of multiple linear regression analysis. For overall subjects, urinary 8-OHdG levels were significantly, inversely associated with serum TC levels (β = −0.0015, p < 0.01) and marginally, inversely associated with LDL-C levels (β = −0.0012, p = 0.07) with adjustment for age, sex, smoking status and body mass index. Urinary 8-OHdG levels were not associated with HDL-C levels (β = −0.0010, p = 0.46). There was no significant interaction of sex on the association between urinary 8-OHdG and cholesterols.

**Table 2:** Regression coefficients in multiple linear regression analyses between serum cholesterols and urinary 8-OHdG

	**n**	**Total cholesterol**			**HDL-cholesterol**			**LDL-cholesterol**		
		**β**	**S.E.**	**p**	**β**	**S.E.**	**p**	**β**	**S.E.**	**p**
Total subjects	503	−0.0015	0.0006	0.01	−0.0010	0.0013	0.46	−0.0012	0.0007	0.07
Men	294	−0.0015	0.0006	0.02	−0.0014	0.0016	0.39	−0.0009	0.0007	0.18
Smoker	134	−0.0017	0.0008	0.04	−0.0029	0.0023	0.22	−0.0007	0.0008	0.37
Non-Smoker	160	−0.0015	0.0010	0.15	−0.0008	0.0021	0.70	−0.0012	0.0012	0.31
Women	209	−0.0022	0.0012	0.08	−0.0004	0.0025	0.86	−0.0030	0.0015	0.04
Age < 48	146	−0.0040	0.0015	0.01	−0.0052	0.0029	0.08	−0.0041	0.0019	0.04
Age ≥ 48	63	−0.0014	0.0019	0.47	0.0033	0.0044	0.46	−0.0027	0.0021	0.22

*8-OHdG* 8-hydroxydeoxyguanosine, *S.E* standard error, *HDL* high-density lipoprotein, *LDL* low-density lipoprotein.

All analyses were adjusted for sex, age, body mass index and smoking status.

LDL-cholesterol was estimated by applying the Friedewald’s formula (LDL cholesterol = total cholesterol - HDL cholesterol - trigriceride/5) only among subjects with fasting blood samples (n = 481).

Subgroup analysis showed that the inverse association with TC was statistically significant among smoking men (β = −0.0017, p < 0.05) and among women aged less than 48 years (β = −0.0040, p < 0.01), but not among non-smoking men (β = −0.0015, p = 0.15) and among women aged 48 years or older (β = −0.0014, p = 0.47). In women, urinary 8-OHdG levels were significantly, inversely associated with LDL-C (β = −0.0030, p < 0.05). Moreover, a marginally significantly, inversely association between with HDL-C and urinary 8-OHdG levels was observed among women aged less than 48 years (β = −0.0052, p = 0.08).

**Figure 2:**
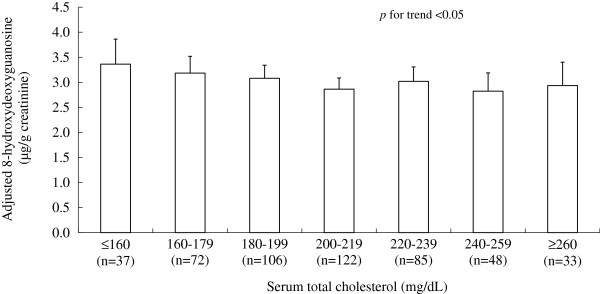
**Mean urinary 8-hydroxydeoxyguanosine (8-OHdG) levels by serum total cholesterol concentrations.** Bar indicates geometric means levels. Error bar indicates upper limit of 95% confidence interval. *Adjusted for age, sex, body mass index and smoking status.

Figure 2 shows mean urinary 8-OHdG by TC levels with adjustment for age, sex, BMI and smoking status. There was a decreasing trend of 8-OHdG with increasing TC (up to 219 mg/dL). The multivariate adjusted means (standard errors) of urinary 8-OHdG levels from the lowest to the highest group of serum TC levels were 3.36 (0.50), 3.19 (0.34), 3.08 (0.26), 2.86 (0.22), 3.02 (0.29), 2.82 (0.37), 2.94 (0.46) (p for trend *<* 0.05). Subjects with TC levels of <160 mg/dL had a 17.4% higher adjusted mean of 8-OHdG than did those with TC levels of 200-219 mg/dL. Results were virtually unchanged after adding other covariates, including alcohol drinking, physical activity, job-title and hypertension status.

## Discussion

We hypothesized that oxidative DNA damage is increased in persons with low cholesterol levels. In the present study of a healthy working population, we found a statistically significant inverse association between serum TC and urinary 8-OHdG concentrations even after adjustment of confounding factors. Subgroup analysis showed that the inverse association was more pronounced in smoking men and younger women. This is one of few epidemiological studies which examined the association between TC levels and oxidative DNA damage [12,13].

Available data on associations between circulating cholesterol levels and markers of oxidative DNA damage are limited and conflicting. In a study of 677 middle-aged city officers, urinary 8-OHdG concentrations were statistically, positively associated with LDL-C, but not with TC [12]. In another study among 90 non-smokers who participated in a health program, a weak non-significant inverse association was observed between TC and urinary 8-OHdG levels (r = −0.155) [13]. In measuring urinary 8-OHdG, these studies used ELISA method, which is less accurate than HPLC method [17].

In the present study, a significant inverse association between TC and urinary 8-OHdG levels was found among smokers (β = −0.0017, p < 0.01) but not among non-smokers (β = −0.0015, p = 0.15) in men. However, the estimated slope (β) of smokers was similar to that of nonsmokers. In women, the association was more apparent among women aged less than 48 years (β = −0.0040, p < 0.01) than those aged 48 years or older (β = −0.0014, p = 0.47). This result may suggest that menopausal status may modify the association between circulating TC and oxidative stress. Such differential association has also been observed in epidemiological studies on cancer. For instance, low serum TC was associated with an increased risk of breast cancer among pre-menopausal women, but not among post-menopausal women [18].

Biological mechanism underlying the association between lower serum TC and higher oxidative DNA damage remains unclear. Cholesterol is an important structural lipid for maintaining cell functions. In addition, cholesterol plays an important role in the absorption of lipid-soluble vitamins including vitamin E, the major membrane-bound antioxidant, and controls the flow of these vitamins in and out of cell membranes [19,20]. Persons with low cholesterol levels may thus have a decreased antioxidant capacity, leading to increased oxidative DNA damage.

In women aged less than 48 years, urinary 8-OHdG levels were significantly, inversely associated with LDL-C (β = −0.0041, p < 0.05), and marginally significantly associated with HDL-C levels (β = −0.0052, p = 0.08). Plausible mechanisms underlying these associations are unclear, but the following has been suggested. HDL-C can prevent oxidation of LDL-C [21]. Thus, LDL-C derived oxidized stress might be increased among subjects with lower levels of HDL-C. Meanwhile, LDL-C level itself is not associated with oxidized LDL-C level [22]. Although LDL-C might be partially oxidized, most of the remaining unoxidized LDL-C might play a protective role against oxidative stress.

Major strengths of the present study include high response rate (91%), control of known background and lifestyle factors associated with oxidative DNA damage, and use of reliable technique (HPLC) in measuring biomarker of oxidative DNA damage [17]. Limitations of the study also warrant mention. The cross-sectional design of this study does not allow us to infer causality. Because study participants were employees of municipal offices in Japan, the present findings may not be applicable to populations with different background. Finally, the sample size of this study may not be sufficient to detect a modest association.

In conclusion, serum TC levels were inversely associated with urinary 8-OHdG concentrations in a healthy working population. This finding suggests that oxidative DNA damage is increased in persons with low cholesterol levels, and thus may support a link of low TC to carcinogenesis.

## Abbreviations

8-OHdG: 8-hydroxydeoxyguanosine; HPLC: The high performance liquid chromatography; BMI: Body mass index; TC: Total cholesterol; LDL-C: Low-density lipoprotein cholesterol; HDL-C: High-density lipoprotein cholesterol.

## Competing interests

The authors declare that they have no competing interest.

## Authors’ contributions

TM, HKA, MS and KK developed study design. TM, AN and AH collected data. HKI, AN and TM performed statistical analysis and prepared draft version of the manuscript. All authors critically revised the manuscript. All authors read and approved the final manuscript.

## References

[B1] Hiatt RA Fireman BH Serum cholesterol and the incidence of cancer in a large cohort J Chronic Dis 1986 39 861 870 10.1016/0021-9681(86)90034-2 3793838

[B2] Schuit AJ Van Dijk CE Dekker JM Schouten EG Kok FJ Inverse association between serum total cholesterol and cancer mortality in Dutch civil servants Am J Epidemiol 1993 137 966 976 8317454 10.1093/oxfordjournals.aje.a116769

[B3] Knekt P Reunanen A Aromaa A Heliovaara M Hakulinen T Hakama M Serum cholesterol and risk of cancer in a cohort of 39,000 men and women J Clin Epidemiol 1988 41 519 530 10.1016/0895-4356(88)90056-X 3290396

[B4] Jafri H Alsheikh-Ali AA Karas RH Baseline and on-treatment high-density lipoprotein cholesterol and the risk of cancer in randomized controlled trials of lipid-altering therapy J Am Coll Cardiol 2010 55 2846 2854 10.1016/j.jacc.2009.12.069 20579542

[B5] Alsheikh-Ali AA Trikalinos TA Kent DM Karas RH Statins, low-density lipoprotein cholesterol, and risk of cancer J Am Coll Cardiol 2008 52 1141 1147 10.1016/j.jacc.2008.06.037 18804740

[B6] Eichholzer M Stahelin HB Gutzwiller F Ludin E Bernasconi F Association of low plasma cholesterol with mortality for cancer at various sites in men: 17-y follow-up of the prospective Basel study Am J Clin Nutr 2000 71 569 574 10648273 10.1093/ajcn/71.2.569

[B7] Rose G Blackburn H Keys A Taylor HL Kannel WB Paul O Reid DD Stamler J Colon cancer and blood-cholesterol Lancet 1974 1 181 183 4129874 10.1016/s0140-6736(74)92492-1

[B8] Okamura T Tanaka H Miyamatsu N Hayakawa T Kadowaki T Kita Y Nakamura Y Okayama A Ueshima U The relationship between serum total cholesterol and all-cause or cause-specific mortality in a 17.3-year study of a Japanese cohort Atherosclerosis 2007 190 216 223 10.1016/j.atherosclerosis.2006.01.024 16529754

[B9] Wannamethee G Shaper AG Whincup PH Walker M Low serum total cholesterol concentrations and mortality in middle aged British men BMJ 1995 311 409 413 10.1136/bmj.311.7002.409 7640584 PMC2550486

[B10] Ames BN Endogenous DNA damage as related to cancer and aging Mutat Res 1989 214 41 46 10.1016/0027-5107(89)90196-6 2671700

[B11] Loft S Vistisen K Ewertz M Tjonneland A Overvad K Poulsen HE Oxidative DNA damage estimated by 8-hydroxydeoxyguanosine excretion in humans: influence of smoking, gender and body mass index Carcinogenesis 1992 13 2241 2247 10.1093/carcin/13.12.2241 1473230

[B12] Sakano N Wang DH Takahashi N Wang B Sauriasari R Kanbara S Sato Y Takigawa T Takaki J Ogino K Oxidative stress biomarkers and lifestyles in Japanese healthy people J Clin Biochem Nutr 2009 44 185 195 10.3164/jcbn.08-252 19308273 PMC2654475

[B13] Miyamoto M Kotani K Ishibashi S Taniguchi N The relationship between urinary 8-hydroxydeoxyguanosine and metabolic risk factors in asymptomatic subjects Med Princ Pract 2011 20 187 190 10.1159/000319774 21252578

[B14] Kasai H Svoboda P Yamasaki S Kawai K Simultaneous determination of 8-hydroxydeoyguanosine, a marker of oxidative stress, and creatinine, a standardization compound, in urine Ind Health 2005 43 333 336 10.2486/indhealth.43.333 15895849

[B15] Hori A Mizoue T Kasai H Kawai K Matsushita Y Nanri A Sato M Ohta M Body iron store as a predictor of oxidative DNA damage in healthy men and women Cancer Sci 2010 101 517 522 10.1111/j.1349-7006.2009.01394.x 19895603 PMC11158582

[B16] Kono S Sunagawa Y Higa H Sunagawa H Age of menopause in Japanese women: trends and recent changes Maturitas 1990 12 43 49 10.1016/0378-5122(90)90059-F 2333036

[B17] Division of Health Promotion and Nutrition Annual report of the national nutrition survey in 2004 2006 Tokyo, Japan: Tokyo Daiichi Publishing Co

[B18] Kelsey JL Berkowitz GS Breast cancer epidemiology Cancer Res 1988 48 5615 5623 3048646

[B19] Burton GW Ingold KU Vitamin E as an in vitro and in vivo antioxidant Ann N Y Acad Sci 1989 570 7 22 10.1111/j.1749-6632.1989.tb14904.x 2698111

[B20] Stahl W van der Berg H Arthur J Bast A Dainty J Faulks RM Gatner C Haenen G Hollman P Holst B Kelly FJ Polidori MC Bioavairability and metabolism Mol Aspects Med 2002 23 39 100 10.1016/S0098-2997(02)00016-X 12079770

[B21] Navab M Anantharamaiah GM Reddy ST Van Lenten BJ Fogelman AM HDL as a biomarker, potential therapeutic target, and therapy Diabetes 2009 58 2711 2717 10.2337/db09-0538 19940234 PMC2780869

[B22] Inoue T Inoue K Maeda H Takayanagi K Morooka S Immunological response to oxidized LDL occurs in association with oxidative DNA damage independently of serum LDL concentrations in dyslipidemic patients Clin Chim Acta 2001 305 115 121 10.1016/S0009-8981(00)00426-5 11249930

